# PROFESSIONAL CONSENSUS ON UK NATIONAL STATEMENTS OF BEST PRACTICE FOR WAYS OF WORKING TO DELIVER ORTHOTIC INTERVENTIONS AFTER STROKE: AN EDELPHI STUDY

**DOI:** 10.2340/jrm.v58.44360

**Published:** 2026-03-04

**Authors:** Miriam GOLDING-DAY, Shirley THOMAS, Phillip WHITEHEAD, Jane HORNE, Marion WALKER

**Affiliations:** 1 Centre of Rehabilitation and Ageing Research, The University of Nottingham, Nottingham; 2 School for Business and Society, University of York, York, UK

**Keywords:** stroke rehabilitation, orthotics intervention, -professional consensus, best practice

## Abstract

**Objective:**

To reach consensus on statements of best practice for ways of working to deliver orthotic interventions after stroke among expert professionals in the UK involved in the delivery of orthotic intervention to patients after stroke.

**Design:**

A 2-round modified electronic Delphi exercise (eDelphi).

**Subjects:**

Thirty-two orthotic professionals with 2 years’ or more experience of delivering orthotic intervention within stroke rehabilitation, from varied geographical locations and experience levels participated in the eDelphi.

**Methods:**

For the eDelphi exercise, 65 statements of best practice were assessed by participants. A 7-point Likert scale was used to determine agreement with statements. A consensus threshold of 75% was pre-determined in line with other studies.

**Results:**

After the first round, consensus was reached for 62 of the statements. All statements had 75% or above agreement. An 87.5% retention rate was maintained between rounds. After the second round 64 statements of best practice achieved 75% consensus.

**Conclusion:**

Overall consensus 94.3% was achieved on the first UK-wide professionally agreed statements of best practice detailing the optimal ways of working when delivering orthotic interventions to enhance rehabilitation outcomes and reduce complications for stroke survivors.

## LAY ABSTRACT

Stroke survivors often have a need for supportive devices called orthotics to help them with standing, walking, and completing daily tasks. These orthotics are provided with the support of a specialist clinician called an orthotist. In the UK, these clinicians are not included in the main stroke rehabilitation team as standard and there are no guidelines for stroke clinical services to follow to help inform how and when orthotists should be involved with a stroke survivor’s care. Statements of best practice are a resource that clinical services, clinicians, and patients can use to guide their clinical care. The primary aim of this project was to develop UK-wide statements of best practice on the optimum ways of working to deliver orthotic intervention after stroke. This study is the culmination of several pieces of research, which ended in reaching a professional agreement with orthotists across the UK on what these statements of best practice should contain. This exercise is called a Delphi and is a tried and tested means of reaching agreement on a healthcare intervention.

Stroke is a leading cause of mortality and disability worldwide and the primary cause of adult disability in the UK (1, 2). Disability due to reduced mobility and gait dysfunction are amongst the most reported physical effects of stroke (1), contributing to difficulties with standing, walking, and the ability to perform daily activities (3, 4). To address these disabilities, many stroke survivors benefit from rehabilitation therapies designed to promote mobility, aid independence, and prevent secondary complications (5). Orthotic intervention is a type of rehabilitation therapy that applies biomechanical principles to help stroke survivors with motor dysfunction and improve the safety and efficiency of standing and walking. Within orthotic intervention therapy an external device, “an orthosis”, is often prescribed to be worn by the patient to help them compensate for any bodily impairments and support their residual function in the neuro-muscular and skeletal systems (6). These devices can therefore offer a useful intervention for stroke survivors to aid in their timely rehabilitation and recovery (7). The number of people using orthotic devices after stroke is unknown but a report by the Foundation for Assistive Technology states that there are approximately 1.2 million orthotic users in England, UK alone (8). A further report asserts the figure may be closer to 2 million (9).

Whilst orthotic interventions are considered a useful adjunct to physical therapies after stroke (5, 10), there is widespread variation in the delivery of orthotic intervention both internationally and at a local level (11, 12). It was identified in 2004 at the “international consensus event on the orthotic management of stroke patients” that there is no agreed “best practice” for identification of patients for whom orthotic fitting would be appropriate, design of the orthosis, and timing of orthotic intervention after stroke (11). The primary output from this event was the first internationally agreed consensus evidence on this topic. Internationally there has been guidance published subsequent to the consensus event detailing optimal approaches to delivering orthotic interventions after stroke (13, 14) and a statement on Ankle–Foot Orthosis use after stroke has been published for Scotland (15). However, no national consensus agreement, statements, or guidelines have been produced for the UK, indicating a need for further research in this area to establish a UK-specific consensus.

As a tool to support clinical practice, statements of best practice are intended to offer guidance to clinicians and commissioners as well as enabling patients to advocate for their own care. The method for which they are developed is therefore important to foster acceptance and ownership of the resulting statements. A modified electronic Delphi (eDelphi) was used to provide a rigorous means of gaining professional consensus as well as taking a participative approach. Delphi is a well-established approach to identifying consensus on a topic, allowing for reflection and facilitating nuance. Commonly used in healthcare research to generate important contributions to the evidence base, Delphi works to conceptualize the primary resource of the expert clinicians, their knowledge, and professional expertise. Before beginning the eDelphi, a process of statement development was undertaken through systematic review (16), national expert (12), and multi-professional surveys (17), as well as stakeholder focus-group consultation. The aim of this study was to refine and finalize statements of best practice through professional consensus on the optimal ways of working when delivering orthotic interventions after stroke. This paper details the process of reaching orthotist consensus via eDelphi on the content and composition of the statements of best practice. These are the first professionally agreed consensus based statements of best practice developed in the UK, detailing the optimal ways of working when delivering orthotic interventions to enhance rehabilitation outcomes and reduce complications for stroke survivors.

## METHODS

**Fig. 1:**
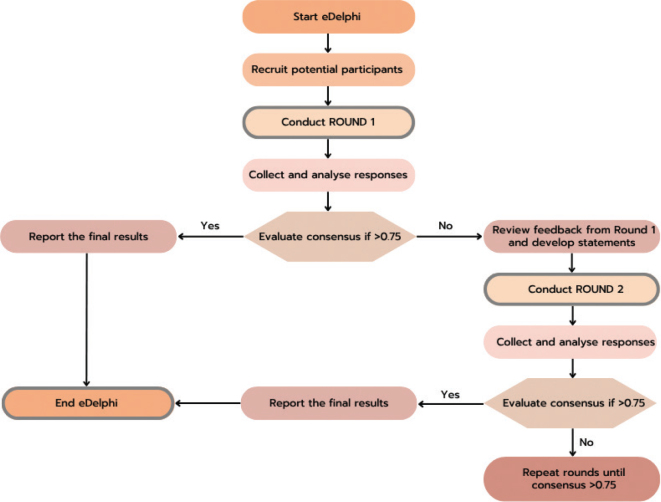
eDelphi consensus flow diagram.

Between 17 April and 31 May 2023 (round 1) and 28 June and 19 July 2023 (round 2) a national eDelphi was completed with orthotic experts within the UK to gain national orthotist consensus on the content and composition of statements of best practice on the delivery of orthotic interventions after stroke. Expert consensus was obtained through a 2-round exercise to produce professionally ratified statements of best practice. For this study, guidance on Conducting and Reporting Delphi Studies (CREDES) was followed as the eDelphi was developed to ensure the study met the requirements to be considered robust and that the results were valid (18). **Fig. 1** presents the eDelphi process followed with defined criteria for when consensus was deemed achieved between rounds, ultimately signifying when the eDelphi was considered complete. A pre-defined consensus threshold was chosen in consistency with other eDelphi studies as described by Diamond et al. (19), with 75% the median threshold reported. The protocol for the eDelphi is available upon request.

### Recruitment of participants

Purposive sampling of potential participants was undertaken as an appropriate approach for exploratory research (20). Potential participants were identified membership of the British Association of Prosthetics and Orthotics (BAPO). As the national professional body for orthotists within the UK, BAPO holds the largest database for registered orthotic professionals in the country. In addition, an email invitation was circulated to all members of the National Orthotic Managers Group (NOMaG), which was then cascaded to clinical teams by their NOMaG manager. Eligibility was self-determined and inclusion criteria consisted of:

- Health and Care Professions Council registered orthotist,
- have > 2 years post-qualification experience in stroke rehabilitation,
- English reading comprehension and writing ability.

### Sample size

There is no agreed standard sample size for an eDelphi expert panel. However, between 30 and 50 participants is considered optimum in reaching consensus for a homogeneous group, allowing for adequate saturation of expert knowledge whilst also ensuring data management is practicable (21). Studies have also indicated that recruiting eDelphi participants from a homogeneous group of experts allows for a smaller sample size to produce the same validity of results (22, 23). To take into account attrition and ensure meaningful conclusions could still be drawn from the data, a proposed sample size of 30 participants was agreed upon with the aim of at least 20 participants completing all rounds. A 70% response rate between rounds was aimed for to maintain statistical rigour (24), and a smaller sample size has been shown to reduce dropout rate (25).

### Development of the eDelphi statements

Proposed statements for the first round of the eDelphi were generated using a convergence triangulation technique as described by Farmer et al. (26), using data generated from our previous studies (12, 16, 17). The themes identified using the coding matrix were tailored to form proposed statements of best practice. This process was completed in co-production with independent clinical and Patient and Public Involvement (PPI) expertise to align the methods and findings of the research with the experiences and needs of orthotic and stroke clinicians and patients who deliver and receive the intervention being investigated (27). The proposed statements were clustered into themes, which were agreed in a co-production meeting with members of the British Association of Prosthetics and Orthotics Research Committee. The individual statements are categorized with others into 1 of the 5 themes and presented as such within the finalized document. Before distribution, the eDelphi round 1 was piloted by independent clinical colleagues and the British Association of Prosthetics and Orthotics Research Committee. Each statement from round 1 of the eDelphi is presented in **Table I**.

**Table I:** eDelphi round 1 statements

1.	The orthotist is an important member of the stroke rehabilitation clinical team
2.	All patients who have experienced a stroke affecting motor control would benefit from having an orthotist assessment
3.	Where an orthotist is not embedded within the stroke clinical unit, regular orthotist contact is important to facilitate timely assessment and treatment
4.	Regular orthotist contact with the stroke unit is important to facilitate knowledge exchange, advice giving, and provide training
5.	The orthotist should lead the orthotics assessment and any subsequent orthotics provision for stroke survivors
6.	When not embedded within a stroke clinical unit, please select how regularly you think the orthotist should be in attendance to assess and treat patients*
7.	Consistency of approach between orthotists in their assessment and treatment is important to ensure equitable treatment for stroke survivors
8.	Where an orthosis has been prescribed it should be the same orthotist who fits and trials that device with the stroke survivor
9.	The stroke survivor should be reviewed by the same orthotist that prescribed and fitted the orthotic device for consistency and continuity of care
10.	Orthotists should have the opportunity to specialize in specific conditions such as neurological rehabilitation
11.	There should be stroke specific training available to pre-registration and post-registration orthotists to establish an agreed pathway of treatment for stroke survivors to encourage conformity across settings
12.	The orthotist should be involved in the planning, commissioning, and provision of stroke rehabilitation services
*Different answer option from other statements. Participants invited to select from: Every day, 2–3 days a week, Once a week (weekly), Every other week (bi-weekly), Once a month (monthly), Less than once a month.	
13.	Orthotic principles such as biomechanics are important considerations when deciding on a stroke survivor’s rehabilitation treatment plan
14.	Ankle Foot Orthoses (AFOs) should be considered for stroke survivors with mobility problems
15.	Any orthotic provision following stroke should be assessed for and prescribed with the intent of “right first time”
16.	Orthoses prescribed for stroke survivors should be considered a rehabilitation tool in their toolbox
17.	The time between assessment and provision of any orthoses for an early rehabilitation stroke survivor should be less than 14 days
18.	Appropriate orthotic provision for stroke survivors can positively influence stance phase and swing phase limb alignment during mobilizing
19.	Orthoses in early post stroke rehabilitation can be used to increase dosage of therapeutic alignment and intervention when hands-on therapist time is limited or unavailable
20.	Orthotic intervention in early post-stroke treatment can contribute to a 24-hour therapy model of rehabilitation
21.	Early orthotic use ensuring correct alignment and normal movement can assist with neuroplasticity retraining of mobility
22.	Following provision of an orthosis, all stroke survivors should have the option of tuning to ensure the best functionality and usability
23.	Availability of orthoses type (custom, prefabricated, material etc.) should be equitable for all stroke survivors regardless of location
24.	Availability of any orthoses for stroke survivors should not be determined by cost and its prescription should be driven by functional need
25.	Custom and personalized orthoses prescriptions should be considered at the first instance over prefabricated orthoses for stroke survivors
26.	Prefabricated orthoses have a valid place within orthotic provision post stroke but should be managed carefully for appropriateness for each individual
27.	Provision of prefabricated orthoses should be overseen by an orthotist or appropriately trained healthcare professional
28.	Information regarding any provided orthoses should be given in both verbal and written formats to stroke survivors
29.	All stroke survivors with mobility problems should be assessed by an orthotist in a timely and equitable manner
30.	Orthotic provision should be considered as a first-line rehabilitation intervention for stroke survivors with lower limb tone and/or spasticity
31.	Orthotic assessment should be considered for any stroke survivor with secondary complication risk factors such as contracture, even if they are non-mobile
32.	Timely and effective orthotist assessment and orthotic provision can enable a stroke survivor’s independence and improve their mental health
33.	For stroke survivors who are prescribed an orthosis, a routine 6-weeks’ follow-up appointment should be made to review progress and ongoing need
34.	Any orthoses prescribed to a stroke survivor should be reviewed at appropriate time periods within the first 12 months of rehabilitation to assess changing need
35.	An orthotic assessment should be available at any stage of a stroke survivor’s rehabilitation, even if no need was identified early after stroke
36.	Difficulties with speech and/or cognition following stroke should be accommodated in any orthotic assessment and provision
37.	Where present, carers should be included in orthotic assessment and provision appointments
38.	An orthosis prescribed for a stroke survivor should be the most cosmetic and unobtrusive in design that is possible, whilst still meeting the individual functional requirements
39.	Appropriate accommodations should be made to enhance compliance of orthoses use when designing an orthotic prescription such as fitting into footwear etc
40.	Orthotics assessment and provision is an integral part of stroke rehabilitation but not a necessity
41.	There should be equity of access to orthotist assessment and orthotic provision across clinical settings (acute hospital, community hospital, and community)
42.	Orthotic assessment and provision for stroke survivors should be prioritized in orthotic service delivery and referrals actioned with urgency
43.	For long-term orthotic users after stroke, a patient or carer should be able to self-refer for further orthotic assessment and review
44.	Relationships between the core orthotic service and the stroke rehabilitation service should be established and nurtured to enhance stroke survivors’ rehabilitation
45.	Orthotic services should be planned, commissioned, and run to provide specialist individualized assessment and treatment for stroke survivors
46.	The core orthotic service should be invested in to facilitate the orthotic delivery and treatment for stroke survivors
47.	An orthotist should be included as a member of the core stroke multidisciplinary rehabilitation (MDT) team
48.	The orthotist should be included in MDT assessments of stroke survivors with motor control problems and contribute to agreed rehabilitation goals and treatments
49.	An orthotic assessment should be conducted as a joint exercise with the specialist skills of the orthotist and therapy staff working together
50.	The use of technology to facilitate joint orthotist assessment such as video evidence or pressure plate technology should be utilized within early stroke rehabilitation
51.	Inclusion of the orthotist within the stroke rehabilitation MDT enhances knowledge exchange and leads to better professional relationships
52.	Orthoses prescription should be undertaken by the orthotist and decided upon using biomechanical principles
53.	Orthotist assessment and provision following a stroke should take a holistic approach seeking to enable tasks of daily living, promote independence, and mental health
54.	Access to orthotist-led orthotic assessment and/or provision should be equitable regardless of where the stroke survivor is located geographically
55.	Scope of practice is an important consideration when an orthotic device is prescribed or fitted by a healthcare professional that is not an orthotist
56.	Assessment by an orthotist for patients presenting with mobility difficulties after a stroke should be conducted within the “early” rehabilitation phase (48 hours in line with early mobilizing recommendations)^1^
57.	Assessment by an orthotist for patients presenting with mobility difficulties after a stroke should be conducted within the “acute” rehabilitation phase (7 days)^2^
58.	Assessment by an orthotist for patients presenting with mobility difficulties after a stroke should be conducted within the “early sub-acute” rehabilitation phase (3 months)^2^
59.	Orthotist assessment and appropriate provision within early gait rehabilitation is an essential mechanism to avoid compensatory patterns being developed such as over-extension at the knee or hip-hiking
60.	Screening tools can be used by other healthcare professionals to help identify if a stroke survivor will benefit from an orthotic assessment and/or provision
61.	Outcome measures should be used to determine the effect of orthotic provision following stroke
62.	Requests from other healthcare professionals for orthotic assessment and/or provision should take the form of referral for assessment rather than a prescription with specified orthoses
63.	Referral for orthotic assessment should be considered for stroke survivors who present with severe impairment where an orthosis may aid in comfort, enable transfer, and assist in care delivery
64.	Stroke services should have a specific referral pathway for orthotic assessment and provision for patients once discharged from the acute hospital setting
65.	There should be training for non-therapy acute and sub-acute clinical staff such as nurses and healthcare assistants on how to correctly put on, remove, and use common types of orthotic device such Ankle–Foot Orthoses

### Definition and calculation of consensus

Consensus was defined *a priori* as ≥ 75% of participants rating a statement within pre-specified agreement or disagreement categories on the Likert scale. This threshold aligns with commonly used cut-offs in Delphi methodology, typically ranging from 70–80% (14).

For each statement, responses on the 7-point Likert scale (1 = strongly disagree, 2 = disagree, 3 = somewhat disagree, 4 = neutral, 5 = somewhat agree, 6 = agree, 7 = strongly agree) were converted to a percentage score using the following formula: *Percentage score = [(Mean Likert score - 1) / (7 - 1)] × 100*

This transformation maps the Likert scale onto a 0–100% scale, where:

- A mean score of 1 (strongly disagree) = 0%.
- A mean score of 4 (neutral) = 50%.
- A mean score of 7 (strongly agree) = 100%.

“Consensus for agreement (inclusion)”: Percentage score ≥ 75% (equivalent to a mean Likert score ≥ 5.5); “Consensus for disagreement (exclusion)”: Percentage score ≤ 25% (equivalent to a mean Likert score ≤ 2.5); and “No consensus”: Percentage scores between 26% and 74% (mean Likert scores between 2.6 and 5.4) were considered to have no consensus and were refined for re-evaluation in subsequent rounds.

For each statement, we calculated the percentage of respondents selecting the 2 highest agreement categories (6–7) and separately the percentage selecting the 2 lowest disagreement categories (1–2). Consensus was determined based on whichever percentage exceeded the 75% threshold. Statements reaching consensus for agreement were to be retained, those reaching consensus for disagreement were to be removed, and those without consensus were carried forward to the next Delphi round with modifications informed by participants’ qualitative feedback.

### Data collection

As a homogeneous clinical group but heterogeneous in geographic location and experience, a modified Delphi in the form of an electronic Delphi (eDelphi) was preferred to collect data from UK practising orthotists. To improve response rate and retention, as well as reduce participant fatigue, a 7-point Likert scale was selected as the means for participant ranking for each statement (29). Use of a consistent scale is considered to have higher validity with the 7-point selected over the 5-point to allow for more nuanced responses between participants. Once each round was complete the data were exported to a Microsoft Excel spreadsheet for analysis (Microsoft Corp, Redmond, WA, USA). This was password protected and no identifiable information was stored.

*Round 1.* For the first round, potential participants received a link to the digital participant information sheet (PIS), consent form, and eDelphi questionnaire. Upon providing consent and submission of demographic details, participants were assigned an identification number so their answers could be tracked between rounds. This identification number was not linked to their email address. Proposed statements to be assessed within the eDelphi were listed on 5 separate pages and grouped with other correlated statements. Sixty-five statements were presented to the expert participants in round 1.

*Round 2.* Participant responses after round 1 were exported from the JISC application and analysed to determine consensus percentage by calculating the mean average for each statement before converting to a percentage. Any statement that did not reach 75% consensus agreement was reviewed and revised. Any statement below 70% consensus agreement was significantly edited or removed. Any statement with over 85% consensus agreement was reviewed and some were edited to use stronger wording. Statements that had 3 or more comments attached to it were reviewed using a simplified thematic analysis (30), to determine commonality across the comments. This was then used to inform the revision of the statement. Participants were then invited to reassess the statements within a second round of the eDelphi. After 75% consensus agreement was reached on all the statements, the eDelphi was considered complete.

### Data analysis

Determination of the consensus percentage of each statement was completed using the Statistical Package for the Social Sciences (SPSS) statistics, version 23 (IBM Corp, Armonk, NY, USA). Descriptive statistics were used to summarize the demographic characteristics of the participants.

### Ethical considerations

Ethical approval for the eDelphi study was obtained from the University of Nottingham Faculty of Medicine and Health Sciences Research Ethics committee on 9 February 2021 (FMHS 156-0121). The eDelphi was developed using the online survey tool JISC (28). Responses were anonymous and stored separately from participants’ contact details, which were used only to send invitation to participate and reminder emails. It was decided that participants would not be presented with their own ranking of statements from previous rounds to reduce the risk of influencing their subsequent response. Similarly, participants were not provided with any indication of how other participants ranked their agreement/disagreement with each statement other than when statements had been altered between rounds.

## RESULTS

Following the circulation of invitations to participate, 32 participants returned responses to round 1 of the eDelphi. In March 2023 there were 1,187 HCPC dual registered prosthetist/orthotists in the UK (31). Thirty-two responses therefore represent 2.7% of the registered workforce within the UK at that time. The time since qualification is not recorded on the HCPC register and so it was not possible to determine the percentage of HCPC registrants who were deemed eligible with 2 years’ or more experience of stroke rehabilitation orthotic practice. However, as a dual registered workforce, approximately half of those on the HCPC register report as practising orthotists, thus indicating a response rate of 5% of registered orthotists in the UK.

All participants were found to be eligible following self-determination of meeting the inclusion criteria. Demographic characteristics of the original 32 participants are presented in **Table II**.

**Table II:** eDelphi participant demographics

	*n* (%)
**Gender**	
Male Female Transgender Other/self-define Prefer not to say	12 (37.5) 20 (62.5) 0 0 0
**Age**	*n* (%)
25–34 years 35–44 years 45–54 years 55–64 years 65 years and over Prefer not to say	17 (53.1) 10 (31.2) 3 (9.4) 2 2 (6.3) 0 0
**Ethnic origin**	*n* (%)
Asian or Asian British Black, Black British, Caribbean, or African Mixed or multiple ethnic groups White Other ethnic group Prefer not to say	2 0 0 29 1 0
**Geographic region of UK currently practising in**	*n* (%)
East of England East Midlands London and surrounding area North-East England Northern Ireland North-West England Scotland South-East England South-West England Wales West Midlands Yorkshire and The Humber Prefer not to say	0 8 (25) 4 (12.5) 1 (3) 2 (6.25) 5 (15.5) 2 (6.5) 1 (3) 1 (3) 0 2 (6.5) 6 (18.75) 0
**Length of time qualified as an orthotist**	*n* (%)
2–5 years 6–10 years 11–20 years 21 years or more	3 (9.5) 16 (50) 9 (28) 4 (12.5)
**Primary employer**	*n* (%)
Public/national health service provider Private contractor for public/national health service Private health service provider Self-employed/locum Higher educational institute Charity/social enterprise Non-governmental organization (NGO) Other (please state)	26 (81.25) 3 (9.5) 2 (6.25) 0 1 (3) 0 0 0
**Predominant clinical setting**	*n* (%)
Large/Acute hospital setting Small district hospital setting Private clinical site not within a hospital Community team treating patients at their usual place of residence (home, care home, school etc.) Research or University site Charity/NGO site Other (please state)	27 (84.5) 3 (9.5) 1 (3) 0 1 (3) 0 0
**Stroke patient clinical caseload**	*n* (%)
More than 5 patients a day Between 2 and 5 patients a day 1 patient a day 1 patient a week 1 patient a month Less frequently than 1 patient a month	0 11 (34.5) 9 (28.125) 9 (28.125) 2 (6.25) 1 (3)
**Time of first assessment after stroke**	*n* (%)
Within 48 hours Within 7 days Within 3 months Within 6 months More than 6 months	0 6 (18.75) 18 (56.25) 4 (12.5) 4 (12.5)

### Participant demographic characteristics

Participants reported working across all regions of the United Kingdom apart from the East of England and Wales. Participants were qualified orthotists who had been practising between 2 and 21 years+, with over 90% qualified for 6 years or more. The mean number of years of experience was 11.3 years (SD ± 5.28). Orthotists practising in the UK are required to have an accredited qualification and be registered with the HCPC. Experience within clinical fields is usually determined by the length of time since qualification and having practised in a field. Within the orthotic profession, it is generally considered that an orthotist is experienced beyond “junior” level after 2 years; 90% (*n* = 29) of the participants had been qualified for 6 years or more. Some 81% of participants reported their predominant employer as a public/national health service provider, namely the NHS.

### Round 1

In round 1, participants were asked to rate 65 statements. Consensus was reached on 95% of the statements (*n* = 62/65) in the first round. Only 3 statements that reached consensus were rated below 80% and no statements had above 75% disagreement. The consensus level percentage for each statement after round 1 is presented in **Table III**. Statements that did not reach 75% agreement are highlighted in orange and statements that had more than 3 feedback comments from participants are highlighted in yellow. Feedback comments were analysed thematically to determine themes and use commonality to further refine the statements before the second round (30). A significant number of comments were received relating to Theme One, Statement 6 and Theme 5, Statements 10, 11, and 12. As a result, the answer options were reduced for Theme One, Statement 6 in an attempt to reach consensus in round 2. Theme 5, Statement 10 was removed; participants indicated this was not a suitable option and statements 11 and 12 were combined into a single statement where participants were asked to select their preferred option.

**Table III:** eDelphi round 1 statement consensus percentage

Statement	Consensus percentage
1. The orthotist is an important member of the stroke rehabilitation clinical team	97%
2. All patients who have experienced a stroke affecting motor control would benefit from having an orthotist assessment	94%
3. Where an orthotist is not embedded within the stroke clinical unit, regular orthotist contact is important to facilitate timely assessment and treatment	97%
4. Regular orthotist contact with the stroke unit is important to facilitate knowledge exchange, advice giving, and provide training	96%
5. The orthotist should lead the orthotics assessment and any subsequent orthotics provision for stroke survivors	96%
6. When not embedded within a stroke clinical unit, please select how regularly you think the orthotist should be in attendance to assess and treat patients* *Different answer option from other statements. Participants invited to select from: Every day, 2–3 days a week, Once a week (weekly), Every other week (bi-weekly), Once a month (monthly), Less than once a month	No consensus
7. Consistency of approach between orthotists in their assessment and treatment is important to ensure equitable treatment for stroke survivors	86%
8. Where an orthosis has been prescribed it should be the same orthotist who fits and trials that device with the stroke survivor	79%
9. The stroke survivor should be reviewed by the same orthotist that prescribed and fitted the orthotic device for consistency and continuity of care	79%
10. Orthotists should have the opportunity to specialize in specific conditions such as neurological rehabilitation	88%
11. There should be stroke specific training available to pre-registration and post-registration orthotists to establish an agreed pathway of treatment for stroke survivors to encourage conformity across settings	92%
12. The orthotist should be involved in the planning, commissioning, and provision of stroke rehabilitation services	90%
13. Orthotic principles such as biomechanics are important considerations when deciding on a stroke survivor’s rehabilitation treatment plan	98%
14. Ankle Foot Orthoses (AFOs) should be considered for stroke survivors with mobility problems	94%
15. Any orthotic provision following stroke should be assessed for and prescribed with the intent of “right first time”	88%
16. Orthoses prescribed for stroke survivors should be considered a rehabilitation tool in their toolbox	93%
17. The time between assessment and provision of any orthoses for an early rehabilitation stroke survivor should be less than 14 days	88%
18. Appropriate orthotic provision for stroke survivors can positively influence stance phase and swing phase limb alignment during mobilizing	99%
19. Orthoses in early post-stroke rehabilitation can be used to increase dosage of therapeutic alignment and intervention when hands-on therapist time is limited or unavailable	95%
20. Orthotic intervention in early post-stroke treatment can contribute to a 24-hour therapy model of rehabilitation	88%
21. Early orthotic use ensuring correct alignment and normal movement can assist with neuroplasticity retraining of mobility	93%
22. Following provision of an orthoses, all stroke survivors should have the option of tuning to ensure the best functionality and usability	94%
23. Availability of orthoses type (custom, prefabricated, material etc.) should be equitable for all stroke survivors regardless of location	99%
24. Availability of any orthoses for stroke survivors should not be determined by cost and its prescription should be driven by functional need	94%
25. Custom and personalized orthosis prescriptions should be considered at the first instance over prefabricated orthoses for stroke survivors	62%
26. Prefabricated orthoses have a valid place within orthotic provision post stroke but should be managed carefully for appropriateness for each individual	91%
27. Provision of prefabricated orthoses should be overseen by an orthotist or appropriately trained healthcare professional	92%
28. Information regarding any provided orthoses should be given in both verbal and written formats to stroke survivors	96%
29. All stroke survivors with mobility problems should be assessed by an orthotist in a timely and equitable manner	96%
30. Orthotic provision should be considered as a first-line rehabilitation intervention for stroke survivors with lower limb tone and/or spasticity	92%
31. Orthotic assessment should be considered for any stroke survivor with secondary complication risk factors such as contracture, even if they are non-mobile	96%
32. Timely and effective orthotist assessment and orthotic provision can enable a stroke survivor’s independence and improve their mental health	98%
33. For stroke survivors who are prescribed an orthosis, a routine 6-week follow-up appointment should be made to review progress and ongoing need	83%
34. Any orthoses prescribed to a stroke survivor should be reviewed at appropriate time periods within the first 12 months of rehabilitation to assess changing need	90%
35. An orthotic assessment should be available at any stage of a stroke survivor’s rehabilitation, even if no need was identified early after stroke	94%
36. Difficulties with speech and/or cognition following stroke should be accommodated in any orthotic assessment and provision	94%
37. Where present, carers should be included in orthotic assessment and provision appointments	97%
38. An orthosis prescribed for a stroke survivor should be the most cosmetic and unobtrusive in design that is possible, whilst still meeting the individual functional requirements	86%
39. Appropriate accommodations should be made to enhance compliance of orthoses use when designing an orthotic prescription such as fitting into footwear etc.	93%
40. Orthotics assessment and provision is an integral part of stroke rehabilitation but not a necessity	52%
41. There should be equity of access to orthotist assessment and orthotic provision across clinical settings (acute hospital, community hospital, and community)	91%
42. Orthotic assessment and provision for stroke survivors should be prioritized in orthotic service delivery and referrals actioned with urgency	82%
43. For long-term orthotic users after stroke, a patient or carer should be able to self-refer for further orthotic assessment and review	90%
44. Relationships between the core orthotic service and the stroke rehabilitation service should be established and nurtured to enhance stroke survivors’ rehabilitation	94%
45. Orthotic services should be planned, commissioned, and run to provide specialist individualized assessment and treatment for stroke survivors	95%
46. The core orthotic service should be invested in to facilitate the orthotic delivery and treatment for stroke survivors	95%
47. An orthotist should be included as a member of the core stroke multidisciplinary rehabilitation (MDT) team	94%
48. The orthotist should be included in MDT assessments of stroke survivors with motor control problems and contribute to agreed rehabilitation goals and treatments	92%
49. An orthotic assessment should be conducted as a joint exercise with the specialist skills of the orthotist and therapy staff working together	93%
50. The use of technology to facilitate joint orthotist assessment such as video evidence or pressure plate technology should be utilized within early stroke rehabilitation	76%
51. Inclusion of the orthotist within the stroke rehabilitation MDT enhances knowledge exchange and leads to better professional relationships	96%
52. Orthoses prescription should be undertaken by the orthotist and decided upon using biomechanical principles	95%
53. Orthotist assessment and provision following a stroke should take a holistic approach seeking to enable tasks of daily living, promote independence, and mental health	98%
54. Access to orthotist-led orthotic assessment and/or provision should be equitable regardless of where the stroke survivor is located geographically	98%
55. Scope of practice is an important consideration when an orthotic device is prescribed or fitted by a healthcare professional that is not an orthotist	97%
56. Assessment by an orthotist for patients presenting with mobility difficulties after a stroke should be conducted within the “early” rehabilitation phase (48 hours in line with early mobilizing recommendations)^1^	69%
57. Assessment by an orthotist for patients presenting with mobility difficulties after a stroke should be conducted within the “acute” rehabilitation phase (7 days)^2^	83%
58. Assessment by an orthotist for patients presenting with mobility difficulties after a stroke should be conducted within the “early sub-acute” rehabilitation phase (3 months)^2^	90%
59. Orthotist assessment and appropriate provision within early gait rehabilitation is an essential mechanism to avoid compensatory patterns being developed such as over-extension at the knee or hip-hiking	93%
60. Screening tools can be used by other healthcare professionals to help identify if a stroke survivor will benefit from an orthotic assessment and/or provision	89%
61. Outcome measures should be used to determine effect of orthotic provision following stroke	95%
62. Requests from other healthcare professionals for orthotic assessment and/or provision should take the form of referral for assessment rather than a prescription with specified orthoses	98%
63. Referral for orthotic assessment should be considered for stroke survivors who present with severe impairment where an orthosis may aid in comfort, enable transfer, and assist in care delivery	98%
64. Stroke services should have a specific referral pathway for orthotic assessment and provision for patients once discharged from the acute hospital setting	95%
65. There should be training for non-therapy acute and sub-acute clinical staff such as nurses and healthcare assistants on how to correctly put on, remove, and use common types of orthotic device such Ankle-Foot Orthoses	95%

1. Langhorne P, Wu O, Rodgers H, Ashburn A, Bernhardt J. A very early rehabilitation trial after stroke (AVERT): a Phase III, multicentre, randomised controlled trial. Health Technol Assess (Rockv) 2017; 21: 1–119. DOI:10.3310/hta21540

2. Bernhardt J, Hayward KS, Kwakkel G, et al. Agreed definitions and a shared vision for new standards in stroke recovery research: the Stroke Recovery and Rehabilitation Roundtable Taskforce. Neurorehabil Neural Repair 2017; 31: 793–799.

### Round 2

Sixty-four statements were circulated for round 2 of the eDelphi on 28/06/2023. An invitation to complete the second round was sent to the 32 expert participants from round 1 with a 3-week deadline to complete. Twenty-eight of the 32 expert participants responded to the second round, an 87.5% retention rate between rounds, ensuring the findings maintained statistical rigour (24). Sixty-three statements reached the pre-defined consensus level of 75% or more with an overall consensus level of 94.31%, indicating a high level of consensus across all the statements when considered in totality. The consensus level percentage for each statement after round 2 is presented in **Table IV**. Statements that were edited between rounds and did not reach as high a consensus as in round 1e are highlighted in blue.

**Table IV:** eDelphi round 2 statement consensus percentage

Statement	SBP section	Consensus percentage
1. The orthotist is an integral member of the stroke rehabilitation clinical team	1(1)	92%
2. All patients who have experienced a stroke affecting motor control should have an orthotist assessment	1(2)	84%
3. Where an orthotist is not embedded within the stroke clinical unit, regular orthotist contact is important to facilitate timely assessment and treatment	1(3)	92%
4. Regular orthotist contact with the stroke unit is important to facilitate knowledge exchange, advice giving, and provide training	1(4)	95%
5. The orthotist should lead the orthotics assessment and any subsequent orthotics provision for stroke survivors who require it	1(5)	95%
6. When not embedded within a stroke rehabilitation unit or community team, the orthotist should attend to assess and treat patients at a minimum of once weekly	1(6)	75%
7. Consistency of approach between orthotists in their assessment and treatment is important to ensure equitable treatment for stroke survivors	1(7)	90%
8. Orthotist continuity of care when an orthosis has been prescribed following stroke is the ideal	1(8)	93%
9. The prescribed orthoses should be reviewed by either the same orthotist or another orthotist from the same team to facilitate collective learning and development of practices	1(9)	92%
10. Orthotists should have the opportunity to specialize in specific conditions such as neurological rehabilitation	1(10)	92%
11. There should be stroke-specific training available to pre-registration and post-registration orthotists to establish an agreed pathway of treatment for stroke survivors to encourage conformity across settings	1(11)	95%
12. The orthotist should be involved in the planning, commissioning, and provision of stroke rehabilitation services	1(12)	91%
13. Orthotic principles such as biomechanics are important considerations when deciding on a stroke survivor’s rehabilitation treatment plan	2(1)	96%
14. Ankle Foot Orthoses (AFOs) should be considered for stroke survivors with mobility problems	2(2)	93%
15. Any orthoses provided following stroke should be done with the intent of “right first time”, to include adjustments and new provision if indicated to address changes in clinical need	2(3)	92%
16. Orthoses prescribed for stroke survivors should be considered a rehabilitation tool in their toolbox	2(4)	93%
17. For new users of an early rehabilitation stroke orthosis, the time between assessment should be less than 14 days	2(5)	93%
18. Appropriate orthotic provision for stroke survivors can positively influence stance phase and swing phase limb alignment during mobilizing	2(6)	98%
19. Orthoses in early post-stroke rehabilitation can be used to increase dosage of therapeutic alignment and intervention when hands-on therapist time is limited or unavailable	2(7)	93%
20. Orthotic intervention in early post stroke treatment can contribute to a 24-hour therapy model of rehabilitation	2(8)	91%
21. Early orthotic use ensuring correct alignment and normal movement can assist with neuroplasticity retraining of mobility	2(9)	93%
22. Following provision of an orthosis, tuning should be optimized to ensure the best functionality and usability for the patient	2(10)	94%
23. Availability of orthosis type (custom, prefabricated, material etc.) should be equitable for all stroke survivors regardless of location	2(11)	98%
24. Provision of any orthosis for stroke survivors should not be determined by cost or convenience and its prescription should be driven by functional need	2(12)	94%
25. Custom and personalized orthosis prescriptions are a preferable prescription in patients with complex gait abnormalities or deformities	2(13)	87%
26. Prefabricated orthoses are a valid and useful orthotic provision post stroke but should be managed carefully for appropriateness for each individual	2(14)	95%
27. Provision of prefabricated orthoses should be overseen by an orthotist or appropriately trained healthcare professional with suitable competencies	2(15)	95%
28. Information regarding any provided orthoses should be given in both verbal and written formats to stroke survivors and their carers	2(16)	97%
29. All stroke survivors with mobility problems should be assessed by an orthotist in a timely and equitable manner	3(1)	90%
30. Orthotic provision should be considered as a first-line rehabilitation intervention for stroke survivors with lower limb tone and/or spasticity	3(2)	91%
31. Orthotic assessment should be considered for any stroke survivor with secondary complication risk factors such as contracture, even if they are non-mobile	3(3)	94%
32. Orthotic assessment and provision should be considered in early gait rehabilitation to avoid compensatory patterns being developed such as over extension at the knee or hip-hiking Statement moved to more appropriate category	3(4)	97%
33. Timely and effective orthotist assessment and orthotic provision can enable a stroke survivor’s independence and improve their mental health	3(5)	96%
34. For stroke survivors who are prescribed an orthosis, a routine follow-up appointment should be made to review progress and ongoing need	3(6)	92%
35. Any orthoses prescribed to a stroke survivor should be reviewed at appropriate time periods within the first 12 months of rehabilitation to assess changing need	3(7)	92%
36. An orthotic assessment should be available at any stage of a stroke survivor’s rehabilitation, even if no need was identified early after stroke	3(8)	95%
37. Difficulties with speech and/or cognition following stroke should be accommodated in any orthotic assessment and provision	3(9)	94%
38. Where present, carers should be included in orthotic assessment and provision appointments	3(10)	95%
39. An orthosis prescribed for a stroke survivor should be the most cosmetic and unobtrusive in design that is possible, whilst still meeting the individual functional requirements and being cost-effective	3(11)	88%
40. Appropriate accommodations should be made to enhance compliance of orthosis use when designing an orthotic prescription such as fitting into footwear etc.	3(12)	94%
41. Appropriate orthotic provision for stroke survivors should positively influence mobility and independence	3(13)	97%
42. Orthotics assessment and provision is an integral part of a stroke rehabilitation service	4(1)	95%
43. There should be equity of access to orthotist assessment and orthotic provision across clinical settings (acute hospital, community hospital, and community)	4(2)	96%
44. Orthotic assessment and provision for stroke survivors should have a degree of priority alongside other more complex patient groups in orthotic service delivery and referrals	4(3)	92%
45. For long-term orthotic users after stroke, a patient or carer should be able to self-refer for further orthotic assessment and review	4(4)	93%
46. Relationships between the core orthotic service and the stroke rehabilitation service should be established and nurtured to enhance stroke survivors’ rehabilitation	4(5)	95%
47. Orthotic services should be planned, commissioned, and run to provide specialist individualized assessment and treatment for stroke survivors	4(6)	97%
48. The core orthotic service should be invested in to facilitate the orthotic delivery and treatment for stroke survivors	4(7)	96%
49. An orthotist should be included as a member of the core stroke multidisciplinary rehabilitation (MDT) team	5(1)	93%
50. The orthotist should be included in MDT assessments of stroke survivors with motor control problems and contribute to agreed rehabilitation goals and treatments	5(2)	93%
51. An orthotic assessment should be conducted as a joint exercise with the specialist skills of the orthotist and therapy staff working together	5(3)	95%
52. The use of technology to facilitate joint orthotist assessment such as video evidence or pressure plate technology can be helpful within early stroke rehabilitation	5(4)	86%
53. Inclusion of the orthotist within the stroke rehabilitation MDT enhances knowledge exchange and leads to better professional relationships	5(5)	96%
54. Orthosis prescription should be decided upon using biomechanical principles led by the orthotist	5(6)	97%
55. Orthotist assessment and provision following a stroke should take a holistic approach seeking to enable tasks of daily living, promote independence, and mental health	5(7)	99%
56. Access to orthotist-led orthotic assessment and/or provision should be equitable regardless of where the stroke survivor is located geographically	5(8)	95%
57. Scope of practice is an important consideration when an orthotic device is prescribed or fitted by a healthcare professional that is not an orthotist	5(9)	97%
58. Assessment by an orthotist for patients presenting with mobility difficulties after a stroke should be conducted within the “acute” (7 days)^1^ or the “early sub-acute” (3 months)^2^ rehabilitation phase after stroke	5(10)	50% - 43%
59. Standardized screening tools are a useful method for other healthcare professionals to help identify if a stroke survivor will benefit from an orthotic assessment and/or provision	5(11)	86%
60. Outcome measures should be used to determine effect of orthotic provision following stroke	5(12)	96%
61. Requests from other healthcare professionals for orthotic assessment and/or provision should take the form of referral for assessment with goal/objective rather than a prescription with specified orthoses	5(13)	98%
62. Referral for orthotic assessment should be considered for stroke survivors who present with severe impairment where an orthosis may aid in comfort, enable transfer, and assist in care delivery	5(14)	97%
63. Stroke services should have a specific referral pathway for orthotic assessment and provision for patients once discharged from the acute hospital setting	5(16)	96%
64. There should be training for non-therapy acute and sub-acute stroke clinical staff such as nurses and healthcare assistants on how to correctly put on, remove, and use common types of orthotic device such as Ankle–Foot Orthoses	5(17)	98%

1. Langhorne P, Wu O, Rodgers H, Ashburn A, Bernhardt J. A very early rehabilitation trial after stroke (AVERT): a Phase III, multicentre, randomised controlled trial. Health Technol Assess (Rockv) 2017; 21: 1–119. DOI: 10.3310/hta21540

2. Bernhardt J, Hayward KS, Kwakkel G, et al. Agreed definitions and a shared vision for new standards in stroke recovery research: the Stroke Recovery and Rehabilitation Roundtable Taskforce. Neurorehabil Neural Repair 2017; 31: 793–799.

As all but one of the statements reached the required consensus level it was agreed no third round was required and the eDelphi was considered complete. The only statement that did not reach expert consensus was Theme Five, Statement 10: “Assessment by an orthotist for patients presenting with mobility difficulties after a stroke should be conducted within: The ‘acute’ rehabilitation phase (7 days), The ‘early sub-acute’ rehabilitation phase (3 months), Other (please state).” Expert participants were split almost 50:50 across 2 options, “acute rehabilitation phase (7 days)” (*n* = 14) and “early sub-acute rehabilitation phase (3 months)” (*n* = 12). The statement was therefore adjusted to incorporate both time options to allow for a degree of interpretation. Any statements that were revised between rounds 1 and 2 of the eDelphi were reviewed and the statement format which held the highest consensus response was included. The full list of statements that reached expert consensus agreement are presented in the “Best Practice Statements: Ways of working to deliver orthotic interventions after stroke”, in Appendix S1.

## DISCUSSION

**Fig. 2:**
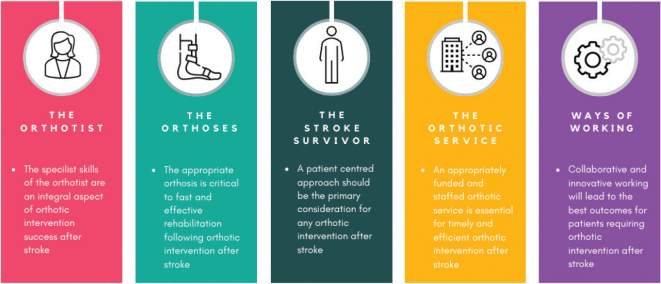
Statements of Best Practice 5 Themes.

An eDelphi was conducted with orthotic experts within the United Kingdom aimed at determining best practice statements for ways of working to deliver orthotic interventions after stroke. This study identified 64 statements of best practice with an overall consensus level of 94.31% achieved. **Fig. 2** shows how the best practice statements are divided into 5 themes: The Orthotist (*n* = 12 statements); The Orthoses (*n* = 16 statements); The Stroke Survivor (*n* = 11 statements); The Orthotic Service (*n* = 7 statements); Ways of Working (*n* = 19 statements).

The themes are considered to be reflective of the wider issues surrounding this area of clinical practice and intended therefore to facilitate the implementation of the statements of best practice within real-world clinical settings.

Professional buy-in on statements of best practice is essential for their eventual adoption and application following their production. Delphi was therefore chosen as a method to begin this professional engagement at the earliest stage of development. As a profession, orthotists are considered the experts in orthotic intervention after stroke and therefore were considered to be best placed to determine best practice and participate as the experts in this Delphi. A diverse Delphi panel helps to provide a broader perspective and therefore generalization of consensus findings. However, a homogeneous expert group can be considered more reliable in a focused Delphi objective (21). Triangulation of previous study findings was completed to formulate the initial proposed statements. This indicates the final statements are embedded in the evidence and based on the perspectives and experiences of different stakeholders including expert orthotists, other health-care professionals, and stroke survivors and carers.

This eDelphi used pre-defined consensus criteria in line with other research and has been reported using CREDES guidance to increase the reliability and transparency of the findings (18). Consensus levels were reported as high in the first round (62/65 statements reached ≥ 75% agreement), which indicates a confirmation of existing views and practice within the profession. This should be taken into consideration when assessing the findings of the Delphi overall as forming a convergence of divergent views. What is indicated within this study is a conformity in theoretical foundation and clinical approach to this area across orthotists practising in the UK, which is in line with other research findings and clinical guidance on the topic.

The findings from this eDelphi are consistent with other international guidance on orthotic provision after stroke (13, 14), and reflect similar themes to those identified within the AFO specific Scottish guidance (15). More widely within stroke rehabilitation, consensus methods as an approach to developing recommendations as a foundation for future evaluative studies are also being promoted internationally (35–37). The statements of best practice that have been refined and agreed within this study can therefore be reviewed in comparison with others produced in this area.

### Strengths and limitations

Considered a strength of the method, the use of an eDelphi facilitated the anonymity of the expert participants. This was considered important when attempting to reach consensus amongst a relatively small expert group such as UK registered orthotists (*n*≈593)^1^ (32). It is, however, important to note that the decision by the survey authors not to provide participants with their previously submitted answers, as a means of retaining confidentiality, is a modification from other Delphi methodology and may have influenced the second-round response. Nevertheless, the overall consensus indicated this choice did not impact on convergence of opinion and, combined with a high retention rate between rounds, the findings and subsequent statements of best practice have a high level of professional confidence and support indicating greater professional buy-in for future implementation.

Orthotic intervention after stroke is only one aspect of post-stroke rehabilitation, with a wide multidisciplinary clinical team working together. As such, the expert opinions of other clinicians, especially those who act as “gatekeepers” to orthotic involvement such as physiotherapists and occupational therapists, could be considered as valuable additions to the voices captured within the development of best practice statements. However, other stakeholder perspectives were included within the previously completed studies from which the proposed statements were developed (12, 16, 17). By triangulating previous study findings to formulate proposed statements, the final statements are embedded in the evidence and based on the perspectives and experiences of stakeholders including expert orthotists, other healthcare professionals, and stroke survivors and carers.

In round 1, participants were asked to provide their demographic characteristics in order to align this work with other research. Every region of the 4 nations of the UK was represented apart from 2: Wales and the East of England. Unlike the previous statement produced on AFO use after stroke, which was developed within Scotland alone (15), these statements can be considered the first expert agreed guidance produced with cross-nation input. The experts who participated in this eDelphi were defined as orthotists with more than 2 years’ post-qualification experience of working with stroke survivors. There is no agreed definition of expert within Delphi literature (33), however Jünger et al. (18) reported that experts in Delphi studies were mostly identified through membership of a professional or stakeholder group, or having specific clinical or academic expertise. A high percentage of participants reported working directly for the NHS as their predominant employer, which was surprising as workforce data report 57% of orthotists in the UK were working for private employers compared with the NHS (34). This potential bias should therefore be noted when considering the generalizability of the study findings as working experiences and practices may be different for direct NHS employees.

The recruitment of expert participants to this eDelphi relied heavily on the invitation circulation to British Association of Prosthetics and Orthotics (BAPO) members and the NHS Orthotic Managers Group (NOMaG). Whilst BAPO is the national professional body for orthotists, their members make up only 54% of HCPC registered practitioners. Moreover, the invitation to an eDelphi is self-selective and is reliant on participants’ willingness to engage with this kind of research activity. This is reflected in that only 5% of the workforce was captured within this Delphi exercise. A potential further bias was noted within the expert participants as they were predominantly white and female; however, this is aligned with the HCPC workforce data from March 2023 (31). The use of online data collection was an efficient methodology, and necessary during the COVID-19 pandemic. It also facilitated participation from a wide geographic area including all nations of the UK except Wales.

### Implications for practice and research: focus on how these statements could be used by clinicians, or patients, while acknowledging feasibility challenges

This paper details the first time expert consensus has been established and presents the first proposed UK-wide statements of best practice detailing the optimal ways of working when delivering orthotic interventions to enhance rehabilitation outcomes and reduce complications for stroke survivors. The statements encompass the different influencing factors that affect the efficient and effective delivery of orthotic intervention after stroke. The expert workforce returned high levels of agreement on what constitutes good practice in this area and have a clear idea as to how to deliver it. The statements produced can be considered representative only of the experience and views of the specific expert participants at a precise snapshot in time. Stroke rehabilitation clinical practice within the UK is directed by a multidisciplinary approach (5, 10) so further engagement and agreement with members of the stroke rehabilitation team as well as the orthotic professional workforce is suggested. Future potential implementation of the statements of best practice produced through this consensus eDelphi should be a future consideration for research in this area, iIn particular the ability to adhere to certain “blue sky” statements that detail perceived optimum care. Examples include Theme 1, Statement 6, which says, “When not embedded within a stroke rehabilitation unit or community team, the orthotist should attend to assess and treat patients at a minimum of once weekly”. Within UK settings, limited resources and relatively small numbers of qualified orthotists (31) mean such a statement would likely be hard to implement in practice. Whilst important to recognize, the purpose of this study was to establish consensus on best practice in this area and in doing so lay out optimum ways of working that are not hindered by real-world practicability. Future research exploring the barrier and facilitators to implementing such recommendations would be of value. This will serve to encourage applicability and acceptability of the statements by those who would be implementing them in clinical settings.

## Supplementary Material


