# Supplementary material for: PROFESSIONAL CONSENSUS ON UK NATIONAL STATEMENTS OF BEST PRACTICE FOR WAYS OF WORKING TO DELIVER ORTHOTIC INTERVENTIONS AFTER STROKE: AN EDELPHI STUDY
Source: J Rehabil Med. 2026 Mar 4;58:44360. doi: 10.2340/jrm.v58.44360 (PMC12969779; doi:10.2340/jrm.v58.44360)
Supplement: Supplementary file 1 [file JRM-58-44360-s1.pdf]

STATEMENTS OF BEST PRACTICE

WAYS OF WORKING TO DELIVER ORTHOTIC INTERVENTIONS AFTER STROKE

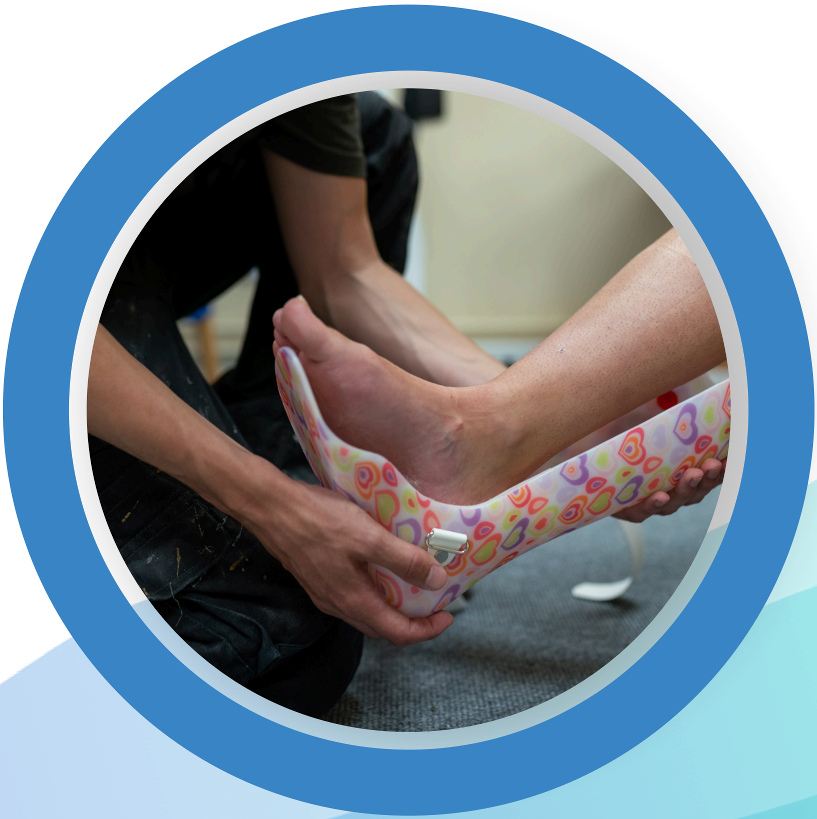

September 2024

AUTHORS

Specialist clinician and project lead

Miriam Golding-Day	Senior Orthotist & Research Fellow, University of Nottingham

Academic advisors

Prof Marion Walker MBE	Occupational Therapist & Emeritus Professor of Stroke Rehabilitation, University of Nottingham
Dr Shirley Thomas	Clinical Pschologist & Associate Professor in Rehabilitation Psychology, University of Nottingham
Dr Phillip Whitehead	Occupational Therapist & Senior Research Fellow, Univesity of York
Dr Jane Horne	Occupational therapist & Senior Research Fellow, University of Nottingham

Clinical advisors

Liz Thomas	Clinical lead and Neurological Specialist Orthotist, Nottingham University Hospital Trust
Paul Charlton	Senior Neurological Specialist Orthotist, Peacocks Medical Group

PPI advisors

Christine Knott	Stroke survivor
Nottingham Stroke Research Partnership Group	Stroke survivor and carer advocacy and support group

Professional advisors

British Association of Prosthetists and Orthotists (BAPO)

NHS Orthotic Managers Group (NOMaG)

Nottingham University Hospitals Orthotic team

CONTENTS

Authors 1

Introduction 3

Best Practice Themes 3

Theme One - The Orthotist 4

Theme Two - The Orthoses 6

Theme Three - The Stroke Survivor 9

Theme Four - The Orthotic Service 11

Theme Five - Ways of Working 12

Glossary 15

References 16

Acknowledgements 22

INTRODUCTION

Whilst orthotic intervention following stroke is often considered an important element of rehabilitation for motor deficit, the optimum content, timing and method of intervention is not agreed. The UK's National clinical guidelines for stroke re-published in 2023 recommend 'access to' orthotic intervention as part stroke rehabilitation services [1], but the mechanisms to facilitate this are unspecified.

We have developed the first professionally agreed statements of best practice for delivering orthotic interventions to support motor recovery after stroke in the UK.

These best practice statements have been developed following the internationally agreed Grading of Recommendations Assessment, Development and Evaluation (GRADE) approach [2]. Each statement was developed against the five criterion for Good Practice Statements [3] and are underpinned by 3 key principles:

- They are intended to guide practice and promote a consistent, cohesive and achievable approach to care. Their aims are realistic but challenging.
- They are primarily intended for use by orthotists and other allied helth professions, but will also be of relevance to the wider clinical team and the patients they treat.
- They are developed where variation in practice exists and seek to establish an agreed approach for practitioners.

These best practice statements will be periodically reviewed, and if necessary, updated in order to ensure they continue to reflect current evidence and professional opinion with regard to best practice.

The statements of best practice themes are divided within this resource into five themes:

THE ORTHOTIST

- The specilist skills of the orthotist are an integral aspect of orthotic intervention success after stroke

Page 4

THE ORTHOSES

- The appropriate orthosis is critical to fast and effective rehabilitation following orthotic intervention after stroke

Page 6

THE STROKE SURVIVOR

- A patient centred approach should be the primary consideration for any orthotic intervention after stroke

Page 9

THE ORTHOTIC SERVICE

- An appropriately funded and staffed orthotic service is essential for timely and efficient orthotic intervention after stroke

Page 11

WAYS OF WORKING

- Collaborative and innovative working will lead to the best outcomes for patients requiring orthotic intervention after stroke

Page 12

THE ORTHOTIST

1. The orthotist is an important member of the stroke rehabilitation clinical team

2. All patients who have experienced a stroke affecting motor control would benefit from having an orthotist assessment

3. Where an orthotist is not embedded within the stroke clinical unit, regular orthotist contact is important to facilitate timely assessment and treatment

4. Regular orthotist contact with the stroke unit is important to facilitate knowledge exchange, advice giving and provide training

5. The orthotist should lead the orthotics assessment and any subsequent orthotics provision for stroke survivors

6. When not embedded within a stroke rehabilitation unit or community team, the orthotist should attend to assess and treat patients at a minimum of once weekly

7. Consistency of approach between orthotists in their assessment and treatment is important to ensure equitable treatment for stroke survivors

THE ORTHOTIST

8 Orthotist continuity of care when an orthosis has been prescribed following stroke is the ideal

9 The prescribed orthosis should be reviewed by either the same orthotist or another orthotist from the same team to facilitate collective learning and development of practices

10 Orthotists should have the opportunity to specialise in specific conditions such as neurological rehabilitation

11 There should be stroke specific training available to pre-registration and post-registration orthotists to establish an agreed pathway of treatment for stroke survivors to encourage conformity across settings

12 The orthotist should be involved in the planning, commissioning and provision of stroke rehabilitation services

THE ORTHOSES

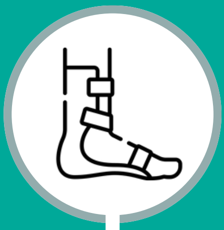

1. Orthotic principles such as biomechanics are important considerations when deciding on a stroke survivors rehabilitation treatment plan

2. Ankle Foot Orthoses (AFOs) should be considered for stroke survivors with mobility problems

3. Any orthosis provided following stroke should be done so with the intent of ‘right first time’, to include adjustments and new provision if indicated to address changes in clinical need

4. Orthoses prescribed for stroke survivors should be considered a rehabilitation tool in their tool box

5. For new users of an early rehabilitation stroke orthosis, the time between assessment and first provision should be less than 14 days

6. Appropriate orthotic provision for stroke survivors can positively influence stance phase and swing phase limb alignment during mobilising

7. Orthoses in early post stroke rehabilitation can be used to increase dosage of therapeutic alignment and intervention when hands on therapist time is limited or unavailable

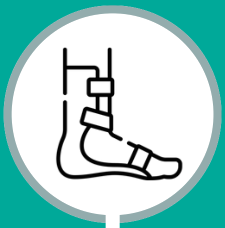

8 Orthotic intervention early post stroke can contribute to a 24 hour therapy model of rehabilitation

9 Early orthotic use ensuring correct alignment and normal movement can assist with neuroplasticity retraining of mobility

10 Following provision of an orthosis post stroke; tuning should be optimised to ensure the best functionality and usability for the patient

11 Availability of orthosis type (custom, prefabricated, material etc.) should be equitable for all stroke survivors regardless of location

12 Availability of any orthosis for stroke survivors should not be determined by cost and its prescription should be driven by functional need

13 Custom and personalised orthoses are a preferable prescription in patients with complex gait abnormalities or deformities

14 Prefabricated orthoses are a valid and useful orthotic provision post stroke but should be managed carefully for appropriateness for each individual

THE ORTHOSES

15 Provision of prefabricated orthoses should be overseen by an orthotist or appropriately trained healthcare professional with suitable competencies

Information regarding any provided orthoses should be given in both verbal and written formats to stroke survivors and their carer 16

THE STROKE SURVIVOR

1 All stroke survivors with mobility problems should be assessed by an orthotist in a timely and equitable manner

2 Orthotic provision should be considered as a first line rehabilitation intervention for stroke survivors with lower limb tone and/or spasticity

3 Orthotic assessment should be considered for any stroke survivor with secondary complication risk factors such as contracture, even if they are non-mobile

4 Orthotic assessment and provision should be considered in early gait rehabilitation to avoid compensatory patterns being developed such as over extension at the knee or hip-hiking

5 Timely and effective orthotist assessment and orthotic provision can enable a stroke survivors independence and improve their mental health

6 For stroke survivors who are prescribed an orthosis, a routine follow-up appointment should be made to review progress and on-going need

7 Any orthoses prescribed to a stroke survivor should be reviewed at appropriate time periods within the first 12 months of rehabilitation to assess changing need

THE STROKE SURVIVOR

8 An orthotic assessment should be available at any stage of a stroke survivors' rehabilitation, even if no need was identified early after stroke

9 Difficulties with speech and/or cognition following stroke should be accommodated in any orthotic assessment and provision

10 Where present, carers should be included in orthotic assessment and provision appointments

11 An orthosis prescribed for a stroke survivor should be the most cosmetic and unobtrusive in design that is possible, whilst still meeting the individual functional requirements and being cost-effective

12 Appropriate accommodations should be made to enhance compliance of orthoses use when designing an orthotic prescription such as fitting into footwear etc.

13 Appropriate orthotic provision for stroke survivors should positively influence mobility and independence

THE ORTHOTIC SERVICE

1 Orthotics assessment and provision should be an integral part of a stroke rehabilitation service

2 There should be equity of access to orthotist assessment and orthotic provision across clinical settings (acute hospital, community hospital and community)

3 Orthotic assessment and provision for stroke survivors should have a degree of priority alongside other more complex patient groups in orthotic service delivery and referrals

4 For long-term orthotic users after stroke, a patient or carer should be able to self-refer for further orthotic assessment and review

5 Relationships between the core orthotic service and the stroke rehabilitation service should be established and nurtured to enhance stroke survivor's rehabilitation

6 Orthotic services should be planned, commissioned and run to provide specialist individualised assessment and treatment for stroke survivors

7 The core orthotic service should be invested in to facilitate the orthotic delivery and treatment for stroke survivors

WAYS OF WORKING

1. An orthotist should be included as a member of the core stroke multi-disciplinary rehabilitation (MDT) team

2. The orthotist should be included in MDT assessments of stroke survivors with motor control problems and contribute to agreed rehabilitation goals and treatments

3. An orthotic assessment should be conducted as a joint exercise with the specialist skills of the orthotist and therapy staff working together

4. The use of technology to facilitate joint orthotist assessment such as video evidence or pressure plate technology can be helpful within early stroke rehabilitation

5. Inclusion of the orthotist within the stroke rehabilitation MDT enhances knowledge exchange and leads to better professional relationships

6. Orthoses prescription should be decided upon using biomechanical principles and led by the orthotist

7. Orthotist assessment and provision following a stroke should take a holistic approach seeking to enable tasks of daily living, promote independence and mental health

8 Access to orthotist led orthotic assessment and/or provision should be equitable regardless of where the stroke survivor is located geographically

9 Scope of practice is an important consideration when an orthotic device is prescribed or fitted by a healthcare professional that is not an orthotist and appropriate competencies must be met

10 Assessment by an orthotist for patients presenting with mobility difficulties after a stroke should be conducted within the 'acute' (7 days) and 'early sub-acute' (3 months) rehabilitation phase after stroke

11 Screening tools can be used by other healthcare professionals to help identify if a stroke survivor will benefit from an orthotic assessment and/or provision

12 Outcome measures should be used to determine effect of orthotic provision following stroke

13 Requests from other health care professionals for orthotic assessment and/or provision should take the form of referral for assessment rather than a prescription with specified orthoses

14 Referral for orthotic assessment should be considered for stroke survivors who present with severe impairment where an orthosis may aid in comfort, enable transfer and assist in care delivery

WAYS OF WORKING

15

Stroke services should have a specific referral pathway for orthotic assessment and provision for patients once discharged from the acute hospital setting

16

There should be training for non-therapy acute and sub-acute stroke clinical staff such as nurses and health care assistants on how to correctly put on, remove and use common types of orthotic device such AFOs

GLOSSARY

Acute setting	A location where healthcare interventions are provided that is within a hospital trust site.
Ankle Foot Orthoses (AFO)	An external brace or splint that fits next to the foot and ankle to control its range of motion and helps to stabilise its position. Patients who have weakness in the ankle dorsiflexor or plantar flexor muscles due to stroke may benefit from the support this device offers in standing and walking. By securing an anatomically correct position, this device can prevent muscle shortening or contracture where muscle imbalance is present after stroke.
Community setting	Any geographical location where healthcare interventions are provided that is not within an acute hospital site.
Contracture	A permanent shortening of a muscle or joint due to loss of motion over time due to abnormal shortening of the soft tissue structures spanning one or more joints. Damage to the brain can result in weakness, decreased motor control, sensation, and spasticity. Through disuse, an affected joint becomes less elastic and stiff and eventually contracted.
Custom orthoses	An orthotic splint or brace that has been made specifically for an individual usually by taking a mould. These usually take longer to provide patients as they have to be manufactured individually and are usually more costly than stock devices.
Orthotist	Orthotists are autonomous registered practitioners who provide gait analysis and engineering solutions to patients with problems of the neuro, muscular and skeletal systems. They are extensively trained at undergraduate level in mechanics, biomechanics and material science along with anatomy, physiology and pathophysiology. It is a protected title within the UK regulated by the Health Care and Professions Council.
Stock orthoses	An orthotic splint or brace that is available 'off-the-shelf' and usually kept in stock at orthotic departments which allows immediate provision. These devices are manufactured to 'normal' size dimensions and are usually less expensive than custom devices. They can be used as a long-term prescription, as a temporary device whilst waiting for more the permanent device to be made, or to test the suitability of a certain kind of support before deciding on a longer-term prescription.

REFERENCES

1. Intercollegiate Stroke Working Party. National Clinical Guideline for Stroke for the UK and Ireland [Internet]. London; 2023. Available from: www.strokeguideline.org
2. Guyatt GH, Oxman AD, Schünemann HJ, Tugwell P, Knottnerus A. GRADE guidelines: A new series of articles in the Journal of Clinical Epidemiology. J Clin Epidemiol. 2011 Apr 1;64(4):380–2.
3. Dewidar O, Lotfi T, Langendam MW, Parmelli E, Saz Parkinson Z, Solo K, et al. Good or best practice statements: proposal for the operationalisation and implementation of GRADE guidance. BMJ Evid Based Med. 2023 Jun 1;28(3):189–96.
4. The Stroke Association. State of the nation: Stroke statistics. London; 2018.
5. Johnson CO, Nguyen M, Roth GA, Nichols E, Alam T, Abate D, et al. Global, regional, and national burden of stroke, 1990–2016: a systematic analysis for the Global Burden of Disease Study 2016. Lancet Neurol. 2019;18(5):439–58.
6. Feigin VL, Forouzanfar MH, Krishnamurthi R, Mensah GA, Connor M, Bennett DA, et al. Global and regional burden of stroke during 1990-2010: Findings from the Global Burden of Disease Study 2010. The Lancet. 2014;383(9913):245–55.
7. Adamson J, Beswick A, Ebrahim S. Is stroke the most common cause of disability? Journal of Stroke and Cerebrovascular Diseases. 2004;13(4):171–7.
8. Department of Health. National Stroke Strategy. London; 2007.
9. National Institute for Health and Care Excellence. Stroke rehabilitation in adults - NICE guideline [NG236]. 2023;(18 Oct). Available from: https://www.nice.org.uk/guidance/ng236
10. Langhorne P, Wu O, Rodgers H, Ashburn A, Bernhardt J. A very early rehabilitation trial after stroke (AVERT): a Phase III, multicentre, randomised controlled trial. Health Technol Assess (Rockv). 2017;21(54):1–119.
11. The Stroke Association. Current, future and avoidable costs of stroke in the UK [Internet]. 2018. Available from: https://www.stroke.org.uk/sites/default/files/costs_of_stroke_in_the_uk_summary_report_0.pdf
12. Saka Ö, McGuire A, Wolfe C. Cost of stroke in the United Kingdom. Age Ageing. 2009;38(1):27–32.
13. Boxer P, Flynn T. Orthotic Pathfinder: A patient-focused strategy and proven implementation plan to improve and expand access to orthotic care services and transform the quality of care delivered. Business Solutions. 2004.
14. The British Association of Prosthetists and Orthotists. Orthotic Treatment: Stroke Rehabilitation. Paisley; 2014.
15. Tamburella F, Moreno JC, Iosa M, Pisotta I, Cincotti F, Mattia D, et al. Boosting the traditional physiotherapist approach for stroke spasticity using a sensorized ankle foot orthosis: A pilot study. Top Stroke Rehabil. 2017;24(6):447–56.
16. Sackley C, Brittle N, Patel S, Ellins J, Scott M, Wright C, et al. The prevalence of joint contractures, pressure sores, painful shoulder, other pain, falls, and depression in the year after a severely disabling stroke. Stroke. 2008;39(12):3329–34.
17. Kwah LK, Harvey LA, Diong JHL, Herbert RD. Half of the adults who present to hospital with stroke develop at least one contracture within six months: An observational study. J Physiother. 2012;58(1):41–7.

18. Teasell RW, McRae MP, Foley N, Bhardwaj A. Physical and functional correlations of ankle-foot orthosis use in the rehabilitation of stroke patients. Arch Phys Med Rehabil. 2001 Aug;82(8):1047–9.
19. Young J, Moss C. Orthotic care needs in a cohort of neurological rehabilitation inpatients. Disabil Rehabil Assist Technol. 2019 Nov;1–5.
20. Momosaki R, Abo M, Watanabe S, Kakuda W, Yamada N, Kinoshita S. Effects of ankle-foot orthoses on functional recovery after stroke: a propensity score analysis based on Japan rehabilitation database. PLoS One. 2015;10(4):e0122688.
21. Cakar E, Durmus O, Tekin L, Dincer U, Kiralp MZ. The ankle-foot orthosis improves balance and reduces fall risk of chronic spastic hemiparetic patients. Eur J Phys Rehabil Med. 2010;46(3):363–8.
22. Tyson S, Sadeghi-Demneh E, Nester C. A systematic review and meta-analysis of the effect of an ankle-foot orthosis on gait biomechanics after stroke. Clin Rehabil. 2013 Oct;27(10):879–91.
23. International Society for Prosthetics and Orthotics. Report of a consensus conference on the orthotic management of stroke patients. Condie E, Cambell J, Martina J, editors. Copenhagen; 2004.
24. Bowers R, Ross K. Best Practice Statement Use of ankle-foot orthoses following stroke. NHS Quality Improvement Scotland. Scotland; 2009.
25. Ramstrand N, Ramstrand S. AAOP State-of-the-Science Evidence Report: The Effect of Ankle-Foot Orthoses on Balance—A Systematic Review. J Prosthet Orthot [Internet]. 2010 Oct 2;P4–23.
26. Guerra Padilla M, Molina Rueda F, Alguacil Diego IM. Effect of ankle-foot orthosis on postural control after stroke: A systematic review. Neurologia. 2014;29(7):423–32.
27. Daryabor A, Kobayashi T, Yamamoto S, Lyons SM, Orendurff M, Akbarzadeh Baghban A. Effect of ankle-foot orthoses on functional outcome measurements in individuals with stroke: a systematic review and meta-analysis. Disabil Rehabil. 2022;44(22):6566–81.
28. Kobayashi E, Hiratsuka K, Haruna H, Kojima N, Himuro N. Efficacy of knee-ankle-foot orthosis on functional mobility and activities of daily living in patients with stroke: A systematic review of care reports. J Rehabil Med. 2022;54.
29. Alexander J, Dawson J, Langhorne P. Dynamic hand orthoses for the recovery of hand and arm function in adults after stroke: A systematic review and meta-analysis of randomised controlled trials. Top Stroke Rehabil. 2022;29(2):114–24.
30. Golding-Day MR, Whitehead PJ, Walker MF. Orthotic intervention following Stroke: A survey of physiotherapist, occupational therapist and orthotist practice and views in the UK. Int J Ther Rehabil. 2022;(June).
31. Shahabi S, Mojgani P, Lankarani KB, Jalali M. The quality of systematic reviews/meta-analyses assessing the effects of ankle-foot orthosis on clinical outcomes in stroke patients: A methodological systematic review. Health Sci Rep. 2023 Mar 1;6(3).
32. Choo YJ, Chang MC. Effectiveness of an ankle–foot orthosis on walking in patients with stroke: a systematic review and meta-analysis. Sci Rep. 2021 Dec 1;11(1).
33. Bernhardt J, Borschmann K, Boyd L, Carmichael ST, Corbett D, Cramer SC, et al. Moving Rehabilitation Research Forward: Developing Consensus Statements for Rehabilitation and Recovery Research. https://doi.org/101177/1545968317724290. 2017 Aug 14;31(8):694–8.

34. Golding-Day MR, Whitehead PJ, Walker MF. Orthotic intervention following Stroke: A survey of physiotherapist, occupational therapist and orthotist practice and views in the UK. Int J Ther Rehabil. 2022;(June).
35. Neumann I, Santesso N, Akl EA, Rind DM, Vandvik PO, Alonso-Coello P, et al. A guide for health professionals to interpret and use recommendations in guidelines developed with the GRADE approach. J Clin Epidemiol. 2016 Apr 1;72:45–55.
36. Dewidar O, Lotfi T, Langendam MW, Parmelli E, Saz Parkinson Z, Solo K, et al. Good or best practice statements: proposal for the operationalisation and implementation of GRADE guidance. BMJ Evid Based Med. 2023 Jun 1;28(3):189–96.
37. Skivington K, Matthews L, Simpson SA, Craig P, Baird J, Blazeby JM, et al. A new framework for developing and evaluating complex interventions: update of Medical Research Council guidance. BMJ. 2021 Sep 30;374.
38. World Health Organisation. International classification of functioning, disability, and health. ICF. 2001;
39. National Institute for Health and Care Excellence. Quality statement 4: Early supported discharge | Stroke in adults [Internet]. National Institute for Health and Care Excellence. NICE; 2016. Available from: https://www.nice.org.uk/guidance/qs2/chapter/quality-statement-4-early-supported-discharge
40. Nikamp C, van der Palen J, Hermens H, Rietman J, Buurke J. The influence of early or delayed provision of ankle-foot orthoses on pelvis, hip and knee kinematics in patients with sub-acute stroke: A randomized controlled trial. Gait Posture. 2018;63:260–7.
41. Portnoy S, Frechtel A, Raveh E, Schwartz I. Prevention of Genu Recurvatum in Poststroke Patients Using a Hinged Soft Knee Orthosis. Physical Medicine & Rehabilitation [Internet]. 2015;7(10):1042–51. Available from: http://ovidsp.ovid.com/ovidweb.cgi?T=JS&PAGE=reference&D=medc1&NEWS=N&AN=25886825
42. Tyson SF, Thornton HA. The effect of a hinged ankle foot orthosis on hemiplegic gait: objective measures and users' opinions. Clin Rehabil. 2001;15(1):53–8.
43. Tyson SF, Kent RM. Effects of an ankle-foot orthosis on balance and walking after stroke: A systematic review and pooled meta-analysis. Arch Phys Med Rehabil. 2013;94(7):1377–85.
44. All-Party Associate Parliamentary Limb Loss Group. Patient led Orthotic services patients charter. 2011;
45. Chockalingam N, Eddison N, Healy A. Cross-sectional survey of orthotic service provision in the UK: does where you live affect the service you receive? BMJ Open. 2019;9(10):28186.
46. Clarke DJ, Forster A. Improving post-stroke recovery: the role of the multidisciplinary health care team. J Multidiscip Healthc. 2015 Sep 22;8:433.
47. Bernhardt J, Hayward KS, Kwakkel G, Ward NS, Wolf SL, Borschmann K, et al. Agreed Definitions and a Shared Vision for New Standards in Stroke Recovery Research: The Stroke Recovery and Rehabilitation Roundtable Taskforce. Neurorehabil Neural Repair [Internet]. 2017 Sep 1 [cited 2023 Jun 23];31(9):793–9. Available from: https://journals.sagepub.com/doi/10.1177/1545968317732668
48. James Lind Alliance. Stroke Rehabilitation and Long-term Care Top 10 Priorities [Internet]. Available from: http://tinyurl.com/2wrvkbx6

49. Golding-Day M, Prince N, Walker M, Thomas S, Horne J, Thomas L. In adult stroke patients does early specialist orthotic intervention improve functional mobility and reduce complications in the lower limb when compared to usual care? PROSPERO. 2020;CRD4202020:1–4.
50. Golding-Day M, Prince N, Thomas S, Horne J, Thomas L, Walker M. Early specialist orthotic interventions for the lower limb in adult stroke patients: a systematic literature review. Journal of the International Foot & Ankle Foundation. 2022;1(9).
51. Wang H, Huang D. The effect of using the AFO in the early time on the walking ability in the hemiplegic stroke patients. Chinese Journal of Rehabilitation Medicine. 2006;21(7):582–4.
52. Nikamp C, Franke J, Schaake L, Rietman J, Buurke J, Prinsen E. A randomized controlled trial on providing ankle-foot orthoses after stroke: Effects on lower limb kinematics of the non-affected side. Gait Posture. 2022 Sep 1;97:S132–3.
53. Nikamp C, Buurke J, van der Palen J, Hermans H. Early or delayed provision of an ankle-foot orthosis in patients with acute and subacute stroke: a randomized controlled trial. Clin Rehabil. 2017;31(6):798–808.
54. Nikamp CDM, Hobbelink MSH, van der Palen J, Hermens HJ, Rietman JS, Buurke JH. The effect of ankle-foot orthoses on fall/near fall incidence in patients with (sub-)acute stroke: A randomized controlled trial. PLoS One. 2019;14(3):e0213538.
55. Nikamp C, Hobbelink M, van der Palen J, Hermens H, Rietman J, Buurke J. A randomized controlled trial on providing ankle-foot orthoses in patients with (sub)acute stroke: Short-term kinematic and spatiotemporal effects and effects of timing. Gait Posture. 2017;55:15–22.
56. Nikamp C, Buurke J, van der Palen J, Hermens H, Rietman J. Six-month effects of early or delayed provision of an ankle-foot orthosis in patients with (sub)acute stroke: a randomized controlled trial. Clin Rehabil. 2017;31(12):1616–24.
57. Wang RY, Lin PY, Lee CC, Yang YR, RY W, PY L, et al. Gait and balance performance improvements attributable to ankle-foot orthosis in subjects with hemiparesis. Am J Phys Med Rehabil. 2007;86(7):556–62.
58. Wang RY, Yen LL, Lee CC, Lin PY, Wang MF. Effects of an ankle-foot orthosis on balance performance in patients with hemiparesis of different durations. Clin Rehabil. 2005;19(1):37–44.
59. Chen C, Yeung K, Wang C, Chu H, Yeh C. Anterior ankle-foot orthosis effects on postural stability in hemiplegic patients. Arch Phys Med Rehabil. 1999;80(12):1587–92.
60. Mojica JA, Nakamura R, Kobayashi T, Handa T, Morohashi I, Watanabe S. Effect of ankle-foot orthosis (AFO) on body sway and walking capacity of hemiparetic stroke patients. Tohoku J Exp Med. 1988;156(4):395–401.
61. Gok H, Kucukdeveci A, Altinkaynak H, Yavuzer G, Ergin S. Effects of ankle-foot orthoses on hemiparetic gait. Clin Rehabil [Internet]. 2003 Mar;17(2):137–9. Available from: http://search.ebscohost.com/login.aspx?direct=true&db=rzh&AN=106694951&site=ehost-live
62. Bleyenheuft C, Caty G, Lejeune T, Detrembleur C. Assessment of the Chignon dynamic ankle-foot orthosis using instrumented gait analysis in hemiparetic adults. Ann Readapt Med Phys. 2008;51(3):154–60.
63. Robinson W, Smith R, Aung O, Ada L. No difference between wearing a night splint and standing on a tilt table in preventing ankle contracture early after stroke: a randomised trial. Aust J Physiother. 2008;54(1):33–8.

64. Leung J, Moseley A, J. L. Impact of ankle-foot orthoses on gait and leg muscle activity in adults with hemiplegia. Physiotherapy. 2003;89(1):39–60.
65. Carse B, Bowers R, Meadows BC, Rowe P. The immediate effects of fitting and tuning solid ankle-foot orthoses in early stroke rehabilitation. Prosthet Orthot Int [Internet]. 2015;39(6):454–62. Available from: http://poi.sagepub.com/content/by/year
66. Wada Y, Otaka Y, Mukaino M, Tsujimoto Y, Shiroshita A, Kawate N, et al. The effect of ankle-foot orthosis on ankle kinematics in individuals after stroke: A systematic review and meta-analysis. PM and R. 2022 Jul 1;14(7):828–36.
67. Daryabor A, Kobayashi T, Yamamoto S, Lyons SM, Orendurff M, Akbarzadeh Baghban A. Effect of ankle-foot orthoses on functional outcome measurements in individuals with stroke: a systematic review and meta-analysis. Disabil Rehabil. 2022;44(22):6566–81.
68. Golding-Day M, Young J, Charlton P, Houston B, Thomas S, Walker M. Orthotist involvement in early gait rehabilitation following stroke: a cross sectional survey of orthotists in the United Kingdom. Prosthet Orthot Int. 2024 Jun 14;6(10–1097).
69. The National Institute for Health and Care Excellence. Stroke rehabilitation in adults: NICE guideline NG236 [Internet]. 2023 [cited 2023 Nov 23]. Available from: https://www.nice.org.uk/guidance/ng236
70. Jakobsen LA, Jørgensen MB. Description of Orthotists Level of Involvement in Early Post Stroke Management in Denmark: A Cross-Sectional Survey [Internet]. Jönköping University; 2021.
71. Eddison N, Healy A, Leone E, Jackson C, Pluckrose B, Chockalingham N. Profile of the UK prosthetic and orthotic workforce and mapping of the workforce for the 21 st century [Internet]. 2023
72. Golding-day M, Houston B, Young J, Charlton P. Early gait re-education training following stroke : a report from the Forward Orthotic Thinking Conference 2021. Synapse. 2021;15–8.
73. Taberna M, Gil Moncayo F, Jané-Salas E, Antonio M, Arribas L, Vilajosana E, et al. The Multidisciplinary Team (MDT) Approach and Quality of Care. Front Oncol [Internet]. 2020 Mar 20 [cited 2024 Apr 28];10:85.
74. The Stroke Association. Lived Experience of Stroke. Chapter 3 - Caring for a stroke survivor: what carers need. London; 2019.
75. Robinson L, Francis J, James P, Tindle N, Greenwell K, Rodgers H. Caring for carers of people with stroke: Developing a complex intervention following the Medical Research Council framework. Clin Rehabil. 2005;19(5):560–71.
76. The Stroke Association. Caring for a stroke survivor [Internet]. Available from: https://www.stroke.org.uk/finding-support/caring-for-a-stroke-survivor
77. Welch VA, Akl EA, Pottie K, Ansari MT, Briel M, Christensen R, et al. GRADE equity guidelines 3: considering health equity in GRADE guideline development: rating the certainty of synthesized evidence. J Clin Epidemiol. 2017 Oct 1;90:76–83.
78. Eddison N, Healy A, Leone E, Jackson C, Pluckrose B, Chockalingam N. The UK prosthetic and orthotic workforce: current status and implications for the future. Hum Resour Health. 2024 Dec 1;22(1):1–9.
79. Bernhardt J, Hayward KS, Dancause N, Lannin NA, Ward NS, Nudo RJ, et al. A Stroke Recovery Trial Development Framework: Consensus-Based Core Recommendations from the Second Stroke Recovery and Rehabilitation Roundtable. Neurorehabil Neural Repair. 2019 Nov 1;33(11):959–69.

80. Skivington K, Matthews L, Simpson SA, Craig P, Baird J, Blazeby JM, et al. Framework for the development and evaluation of complex interventions: gap analysis, workshop and consultation-informed update. Health Technol Assess (Rockv). 2021 Sep 30;25(57):1–132.
81. Leone E, Eddison N, Healy A, Jackson C, Pluckrose B, Chockalingam N. The national profile of the prosthetic and orthotic workforce in the UK: Sociodemographics and employment characteristics. Prosthet Orthot Int. 2024;1–10.
82. Golding-Day M, Young J, Charlton PT. Orthotist involvement in early stroke rehabilitation: An update on research and education work streams. In: British Association of Prosthetists and Orthotists (BAPO) conference. 2021.

ACKNOWLEDGMENTS

The research team would like to acknowledge the contribution to these Statements of Best Practice by the many healthcare professionals, stroke survivors, and carers involved with the development of this resource.

We would like to thank the Stroke Association for funding the research (SA PGF 19\100008), which lead to the development of this resource, and the National Institute for Health Research (NIHR) Applied Research Collaboration East Midlands (ARC EM) for supporting the project and acknowledging it within their portfolio.

The views and recommendations expressed in these Statements of Best Practice are those of the author(s) and not necessarily those of the Stroke Association, National Health Service, National Institute for Health Research, the Department of Health and social care.
